# Family Proximity as a Moderator of Spousal Associations of Depression among Mexican Older Adults

**DOI:** 10.1093/geroni/igab046.2786

**Published:** 2021-12-17

**Authors:** Katie Newkirk, Maria Aranda, Catalina Mourgues-Codern, Ana Quiñones, Rafael Samper-Ternent, Joan Monin

**Affiliations:** 1 Yale University School of Medicine, New Haven, Connecticut, United States; 2 University of Southern California, Los Angeles, California, United States; 3 Oregon Health & Science University, Portland, Oregon, United States; 4 The University of Texas Medical Branch, Galveston, Texas, United States; 5 Yale School of Public Health, New Haven, Connecticut, United States

## Abstract

Depression among older adults is a public health issue, and a large literature highlights the importance of close relationships as both a risk and protective factor for depression. Research in U.S. samples suggests that one spouse’s depressive symptoms can increase their partner’s depressive symptoms, especially for women (Kouros & Cummings, 2010; Tower & Kasl, 1996). Little is known about interpersonal associations in depression, mitigating factors, and the role of gender among older couples in Mexico. This study examined (1) the effects of an individual’s depressive symptoms on their spouse’s symptoms and 2) whether living close to family buffered depression associations using data from the Mexican Health and Aging Study (n=4,071 dyads, age 50+ at initial interview). Depressive symptoms were measured in 2001, 2003, 2012, 2015, and 2018 using a modified 8-item version of the Center for Epidemiologic Studies-Depression Scale. Multilevel modeling was used to fit a dual-intercept growth model (centered at 2012) of husbands’ and wives’ depressive symptoms over time, controlling for age and education. Results showed a partner effect for husbands and wives, such that having a spouse with greater depressive symptoms in 2001 was associated with greater subsequent depressive symptoms, but not with rate of change in symptoms, in 2012. There was also a moderation effect such that the deleterious effect of husbands’ depressive symptoms on wives’ symptoms, as well as rate of increase in symptoms, was higher when family lived nearby, suggesting family may potentially exacerbate depression associations among spouses rather than a buffering them as hypothesized.

